# [2-(2,5-Dichloro­benz­yl)-4-hy­droxy-1,1-dioxo-2*H*-1,2-benzothia­zin-3-yl](phen­yl)methanone

**DOI:** 10.1107/S160053681201481X

**Published:** 2012-04-13

**Authors:** Nazia Sattar, Hamid Latif Siddiqui, Matloob Ahmad, Muhammad Akram, Masood Parvez

**Affiliations:** a Institute of Chemistry, University of the Punjab, Lahore 54590, Pakistan; b Chemistry Department, Govt. College University, Faisalabad, Pakistan; c Department of Chemistry, Universiti Teknologi Malaysia, 81310 UTM Skudai, Johor, Darul Ta’zim, Malaysia; d Department of Chemistry, The University of Calgary, 2500 University Drive NW, Calgary, Alberta, Canada T2N 1N4

## Abstract

In the title mol­ecule, C_22_H_15_Cl_2_NO_4_S, the heterocyclic thia­zine ring adopts a half-chair conformation, with the S and N atoms displaced by 0.343 (5) and 0.402 (5) Å, respectively, on opposite sides of the mean plane formed by the remaining ring atoms. The mol­ecular structure is consolidated by an intra­molecular O—H⋯O hydrogen bond, which generates an *S*(?) ring. In the crystal, the molecules are linked by C—H⋯O interactions into [010] chains.

## Related literature

For background information on the activity of anti-inflammatory and analgesic oxicams, see: Lombardino *et al.* (1971 ▶); Soler (1985 ▶); Carty *et al.* (1993 ▶); Turck *et al.* (1995 ▶); Blackham & Owen (1975 ▶). For the biological activity of benzothia­zine derivatives, see: Zia-ur-Rehman *et al.* (2005 ▶); Ahmad *et al.* (2010 ▶). For the syntheses and crystal stuctures of related benzothia­zine derivatives, see: Ahmad *et al.* (2011 ▶); Aslam *et al.* (2012 ▶).
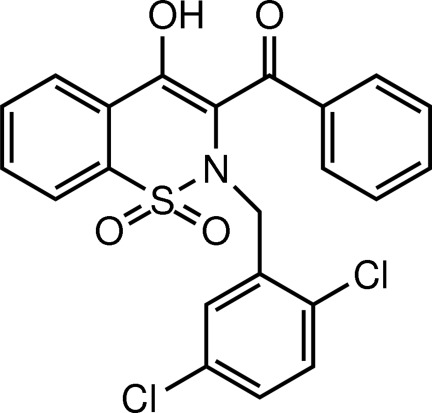


## Experimental

### 

#### Crystal data

C_22_H_15_Cl_2_NO_4_S
*M* *_r_* = 460.32
Monoclinic, 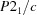

*a* = 12.8172 (5) Å
*b* = 9.9215 (4) Å
*c* = 16.7155 (5) Å
β = 110.511 (2)°
*V* = 1990.89 (13) Å^3^
*Z* = 4
Mo *K*α radiation
μ = 0.46 mm^−1^
*T* = 173 K
0.20 × 0.18 × 0.16 mm

#### Data collection

Nonius KappaCCD diffractometer
Absorption correction: multi-scan (*SORTAV*; Blessing, 1997 ▶) *T*_min_ = 0.913, *T*_max_ = 0.930
16108 measured reflections
4592 independent reflections
3598 reflections with *I* > 2σ(*I*)
*R*_int_ = 0.055

#### Refinement

*R*[*F* ^2^ > 2σ(*F* ^2^)] = 0.057
*wR*(*F* ^2^) = 0.118
*S* = 1.11
4592 reflections
272 parameters
H-atom parameters constrained
Δρ_max_ = 0.33 e Å^−3^
Δρ_min_ = −0.44 e Å^−3^

### 

Data collection: *COLLECT* (Hooft, 1998 ▶); cell refinement: *DENZO* (Otwinowski & Minor, 1997 ▶); data reduction: *SCALEPACK* (Otwinowski & Minor, 1997 ▶); program(s) used to solve structure: *SHELXS97* (Sheldrick, 2008 ▶); program(s) used to refine structure: *SHELXL97* (Sheldrick, 2008 ▶); molecular graphics: *ORTEP-3* (Farrugia, 1997 ▶); software used to prepare material for publication: *SHELXL97*.

## Supplementary Material

Crystal structure: contains datablock(s) global, I. DOI: 10.1107/S160053681201481X/rk2344sup1.cif

Structure factors: contains datablock(s) I. DOI: 10.1107/S160053681201481X/rk2344Isup2.hkl

Supplementary material file. DOI: 10.1107/S160053681201481X/rk2344Isup3.cml

Additional supplementary materials:  crystallographic information; 3D view; checkCIF report

## Figures and Tables

**Table 1:** Hydrogen-bond geometry (Å, °)

*D*—H⋯*A*	*D*—H	H⋯*A*	*D*⋯*A*	*D*—H⋯*A*
C3—H3⋯O4^i^	0.95	2.60	3.310 (4)	132
O3—H3*O*⋯O4	0.84	1.80	2.539 (3)	146

Symmetry code: (i) 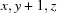.

**Fig. 1.**
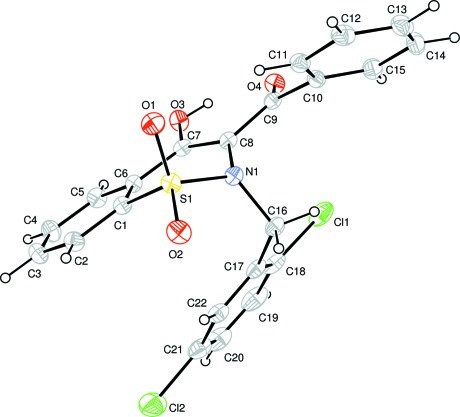
The molecular structure of the title compound with the atom numbering scheme. Displacement ellipsoids are drawn at the 25% probability level. The H atoms are presented as small spheres of arbitrary radius.

**Fig. 2.**
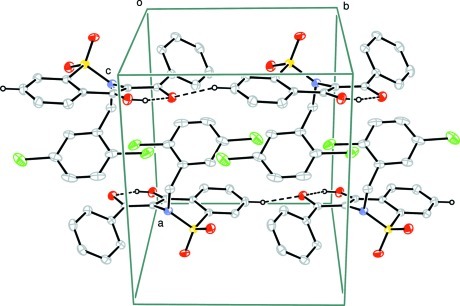
A part of the unit cell showing intermolecular and intramolecular hydrogen bonds (dotted lines) in the crystal structure of the title compound. H atoms non-participating in hydrogen-bonding were omitted for clarity.

## supplementary crystallographic information

### Comment

*Oxicam* is the most recent class of non-steroidal anti-inflammatory drugs (*NSAID*s) and includes molecules that are derived from 1,2-benzothiazine-1,1-dioxide nuclei which are found to be potent anti-inflammatory and analgesic agents, *e.g.*, *piroxicam* (Lombardino *et al.*, 1971), *droxicam* (Soler, 1985), *ampiroxicam* (Carty *et al.*, 1993), *meloxicam* (Turck *et al.*, 1995) and *sudoxicam* (Blackham & Owen, 1975), *etc*. Besides *oxicam*, a large number of benzothiazine derivatives are found to possess anti-microbial (Zia-ur-Rehman *et al.*, 2005) and anti-oxidant activities (Ahmad *et al.*, 2010). As part of our ongoing research we are interested in the synthesis and characterization of novel benzothiazine derivatives (Aslam *et al.*, 2012; Ahmad *et al.*, 2011). In this paper we report the synthesis, molecular and crystal structure of the title compound.

The bond distances and angles in the title compound (Fig. 1) agree well with the corresponding bond distances and angles reported for structures of closely related compounds (Ahmad *et al.*, 2011; Aslam *et al.*, 2012). The heterocyclic thiazine ring adopts a half chair conformation with atoms N1 and S1 displaced by 0.402 (5)Å and 0.343 (5)Å, respectively, on the opposite sides from the mean plane formed by the remaining ring atoms. The dihedral angle between the mean planes of benzene rings C1-C6 and C17-C22 is 31.17 (7)° while the mean planes of the benzene rings C1-C6 and C10-C15 are oriented at 35.09 (9)° with respect to each other. The molecular structure of the title compound is stabilized by intramolecular interactions O3–H3O···O4, C11–H11···N1 and C16–H16A···O2, *etc*, while the crystal packing is consolidated by C3–H3···O4^i^ intermolecular nonclassical hydrogen bonds resulting in chains of molecules lying along the *b*-axis (Fig. 2 and Table 1). Symmetry code: (i) *x*, *y*+1, *z*.

### Experimental

A mixture of 3-benzoyl-4-hydroxy-2*H*-1,2-benzothiazine 1,1-dioxide (1.0 g, 3.32 mmol), aqueous sodium hydroxide (0.26 g, 6.6 mmol) and 2-(bromomethyl)-1,4-dichlorobenzene (0.80 g, 3.32 mmol) in acetone (10 ml) was subjected to ultrasonic irradiation for 20 minutes at 318 K. The reaction mixture was then acidified to pH = 3 by using dilute hydrochloric acid. The precipitates were filtered, washed with excess of distilled water and dried at room temperature to get chrome yellow powder of the title compound (1.38 g, 90.3%). The crystals suitable for X-ray crystallographic analysis were grown from methanol by slow evaporation at room temperature.

### Refinement

The H atoms bonded to C and O atoms were positioned geometrically and refined using a riding model, with O–H = 0.84Å and C–H = 0.95Å and 0.99Å, respectively, for aryl and methylene type H-atoms. The *U*_iso_(H) were allowed at 1.2*U*_eq_(parent atom).

### Crystal data

**Table:**

C_22_H_15_Cl_2_NO_4_S	*F*(000) = 944
*M**_r_* = 460.32	*D*_x_ = 1.536 Mg m^−^^3^
Monoclinic, *P*2_1_/*c*	Mo *K*α radiation, λ = 0.71073 Å
Hall symbol: -P 2ybc	Cell parameters from 8696 reflections
*a* = 12.8172 (5) Å	θ = 1.0–27.5°
*b* = 9.9215 (4) Å	µ = 0.46 mm^−^^1^
*c* = 16.7155 (5) Å	*T* = 173 K
β = 110.511 (2)°	Prism, yellow
*V* = 1990.89 (13) Å^3^	0.20 × 0.18 × 0.16 mm
*Z* = 4	

### Data collection

**Table:**

Nonius KappaCCD diffractometer	4592 independent reflections
Radiation source: fine-focus sealed tube	3598 reflections with *I* > 2σ(*I*)
Graphite monochromator	*R*_int_ = 0.055
ω and φ scans	θ_max_ = 27.6°, θ_min_ = 2.7°
Absorption correction: multi-scan (*SORTAV*; Blessing, 1997)	*h* = −16→16
*T*_min_ = 0.913, *T*_max_ = 0.930	*k* = −12→12
16108 measured reflections	*l* = −21→20

### Refinement

**Table:**

Refinement on *F*^2^	Primary atom site location: structure-invariant direct methods
Least-squares matrix: full	Secondary atom site location: difference Fourier map
*R*[*F*^2^ > 2σ(*F*^2^)] = 0.057	Hydrogen site location: inferred from neighbouring sites
*wR*(*F*^2^) = 0.118	H-atom parameters constrained
*S* = 1.11	*w* = 1/[σ^2^(*F*_o_^2^) + (0.0232*P*)^2^ + 2.3517*P*] where *P* = (*F*_o_^2^ + 2*F*_c_^2^)/3
4592 reflections	(Δ/σ)_max_ = 0.001
272 parameters	Δρ_max_ = 0.33 e Å^−^^3^
0 restraints	Δρ_min_ = −0.44 e Å^−^^3^

### Special details

**Table:**

Geometry. All s.u.'s (except the s.u. in the dihedral angle between two l.s. planes) are estimated using the full covariance matrix. The cell s.u.'s are taken into account individually in the estimation of s.u.'s in distances, angles and torsion angles; correlations between s.u.'s in cell parameters are only used when they are defined by crystal symmetry. An approximate (isotropic) treatment of cell s.u.'s is used for estimating s.u.'s involving l.s. planes.
Refinement. Refinement of *F*^2^ against ALL reflections. The weighted *R*-factor *wR* and goodness of fit *S* are based on *F*^2^, conventional *R*-factors *R* are based on *F*, with *F* set to zero for negative *F*^2^. The threshold expression of *F*^2^ > σ(*F*^2^) is used only for calculating *R*-factors(gt) *etc*. and is not relevant to the choice of reflections for refinement. *R*-factors based on *F*^2^ are statistically about twice as large as those based on *F*, and *R*-factors based on ALL data will be even larger.

### Fractional atomic coordinates and isotropic or equivalent isotropic displacement parameters (Å^2^)

**Table:**

	*x*	*y*	*z*	*U*_iso_*/*U*_eq_	
Cl1	0.52481 (7)	−0.04749 (10)	0.39033 (6)	0.0658 (3)	
Cl2	0.50174 (9)	0.57915 (12)	0.37257 (7)	0.0804 (3)	
S1	0.87675 (5)	0.25684 (7)	0.53887 (4)	0.03478 (16)	
O1	0.98780 (15)	0.2067 (2)	0.56375 (12)	0.0457 (5)	
O2	0.84712 (17)	0.3467 (2)	0.59407 (11)	0.0465 (5)	
O3	0.79566 (17)	0.0186 (2)	0.31269 (11)	0.0418 (5)	
H3O	0.7866	−0.0624	0.3235	0.050*	
O4	0.77251 (15)	−0.18006 (19)	0.40080 (11)	0.0408 (5)	
N1	0.79342 (17)	0.1262 (2)	0.52149 (12)	0.0331 (5)	
C1	0.8421 (2)	0.3280 (3)	0.43643 (16)	0.0339 (6)	
C2	0.8521 (2)	0.4653 (3)	0.42629 (18)	0.0406 (6)	
H2	0.8739	0.5236	0.4744	0.049*	
C3	0.8297 (2)	0.5165 (3)	0.34482 (19)	0.0465 (7)	
H3	0.8365	0.6104	0.3369	0.056*	
C4	0.7977 (3)	0.4316 (3)	0.27565 (18)	0.0483 (7)	
H4	0.7830	0.4676	0.2201	0.058*	
C5	0.7866 (2)	0.2949 (3)	0.28534 (17)	0.0421 (7)	
H5	0.7638	0.2378	0.2366	0.050*	
C6	0.8089 (2)	0.2404 (3)	0.36662 (15)	0.0318 (5)	
C7	0.7989 (2)	0.0952 (3)	0.37903 (15)	0.0326 (6)	
C8	0.7978 (2)	0.0396 (3)	0.45361 (14)	0.0307 (5)	
C9	0.7971 (2)	−0.1051 (3)	0.46462 (16)	0.0334 (6)	
C10	0.8276 (2)	−0.1656 (3)	0.55114 (16)	0.0357 (6)	
C11	0.9160 (2)	−0.1140 (3)	0.61913 (17)	0.0403 (6)	
H11	0.9531	−0.0345	0.6119	0.048*	
C12	0.9498 (2)	−0.1792 (3)	0.69745 (18)	0.0463 (7)	
H12	1.0107	−0.1448	0.7438	0.056*	
C13	0.8954 (3)	−0.2934 (3)	0.70810 (19)	0.0498 (8)	
H13	0.9190	−0.3376	0.7619	0.060*	
C14	0.8066 (3)	−0.3445 (3)	0.6410 (2)	0.0505 (8)	
H14	0.7685	−0.4226	0.6491	0.061*	
C15	0.7735 (3)	−0.2815 (3)	0.56220 (19)	0.0448 (7)	
H15	0.7137	−0.3175	0.5157	0.054*	
C16	0.6823 (2)	0.1408 (3)	0.53043 (16)	0.0376 (6)	
H16A	0.6906	0.1968	0.5813	0.045*	
H16B	0.6560	0.0507	0.5403	0.045*	
C17	0.5954 (2)	0.2036 (3)	0.45391 (16)	0.0396 (6)	
C18	0.5232 (2)	0.1274 (4)	0.38632 (19)	0.0502 (8)	
C19	0.4486 (3)	0.1900 (5)	0.3150 (2)	0.0661 (11)	
H19	0.4009	0.1372	0.2695	0.079*	
C20	0.4435 (3)	0.3273 (5)	0.3101 (2)	0.0685 (11)	
H20	0.3933	0.3699	0.2606	0.082*	
C21	0.5114 (3)	0.4044 (4)	0.37701 (19)	0.0564 (9)	
C22	0.5870 (2)	0.3432 (3)	0.44836 (17)	0.0437 (7)	
H22	0.6336	0.3972	0.4938	0.052*	

### Atomic displacement parameters (Å^2^)

**Table:**

	*U*^11^	*U*^22^	*U*^33^	*U*^12^	*U*^13^	*U*^23^
Cl1	0.0454 (4)	0.0807 (7)	0.0762 (6)	−0.0194 (4)	0.0274 (4)	−0.0311 (5)
Cl2	0.0780 (7)	0.0882 (8)	0.0780 (6)	0.0331 (6)	0.0310 (5)	0.0324 (6)
S1	0.0355 (3)	0.0382 (4)	0.0259 (3)	−0.0025 (3)	0.0048 (2)	−0.0050 (3)
O1	0.0316 (10)	0.0547 (13)	0.0402 (10)	−0.0033 (9)	−0.0006 (8)	−0.0019 (9)
O2	0.0582 (13)	0.0452 (12)	0.0332 (10)	−0.0025 (10)	0.0124 (9)	−0.0114 (9)
O3	0.0569 (12)	0.0388 (11)	0.0303 (9)	−0.0066 (10)	0.0160 (9)	−0.0086 (8)
O4	0.0440 (11)	0.0395 (11)	0.0358 (10)	−0.0041 (9)	0.0102 (8)	−0.0087 (8)
N1	0.0342 (11)	0.0379 (12)	0.0265 (10)	−0.0022 (10)	0.0100 (9)	−0.0041 (9)
C1	0.0311 (13)	0.0375 (15)	0.0310 (13)	−0.0010 (11)	0.0082 (10)	−0.0024 (11)
C2	0.0414 (15)	0.0378 (15)	0.0399 (15)	−0.0015 (12)	0.0108 (12)	−0.0062 (12)
C3	0.0529 (18)	0.0349 (16)	0.0515 (17)	0.0000 (13)	0.0178 (14)	0.0047 (13)
C4	0.0578 (19)	0.0464 (18)	0.0389 (15)	−0.0002 (15)	0.0146 (14)	0.0081 (13)
C5	0.0469 (16)	0.0474 (17)	0.0304 (13)	−0.0005 (13)	0.0117 (12)	−0.0024 (12)
C6	0.0305 (12)	0.0366 (14)	0.0276 (11)	−0.0021 (11)	0.0092 (10)	−0.0020 (11)
C7	0.0281 (12)	0.0398 (15)	0.0290 (12)	−0.0016 (11)	0.0088 (10)	−0.0074 (11)
C8	0.0276 (12)	0.0380 (14)	0.0246 (11)	−0.0008 (11)	0.0066 (9)	−0.0035 (10)
C9	0.0274 (12)	0.0388 (15)	0.0337 (13)	−0.0021 (11)	0.0103 (10)	−0.0028 (11)
C10	0.0379 (14)	0.0354 (14)	0.0349 (13)	0.0042 (12)	0.0140 (11)	0.0028 (11)
C11	0.0391 (15)	0.0417 (16)	0.0387 (14)	0.0041 (12)	0.0119 (12)	0.0034 (12)
C12	0.0454 (16)	0.0536 (19)	0.0365 (15)	0.0096 (14)	0.0102 (13)	0.0026 (13)
C13	0.062 (2)	0.0496 (19)	0.0413 (16)	0.0147 (16)	0.0226 (15)	0.0116 (14)
C14	0.067 (2)	0.0375 (17)	0.0579 (19)	0.0017 (15)	0.0351 (17)	0.0071 (14)
C15	0.0504 (17)	0.0423 (17)	0.0449 (16)	−0.0002 (13)	0.0206 (14)	−0.0016 (13)
C16	0.0365 (14)	0.0490 (17)	0.0306 (13)	−0.0006 (12)	0.0156 (11)	−0.0037 (12)
C17	0.0292 (13)	0.0628 (19)	0.0296 (13)	0.0001 (13)	0.0138 (11)	−0.0029 (12)
C18	0.0313 (14)	0.079 (2)	0.0444 (16)	−0.0037 (15)	0.0190 (13)	−0.0138 (16)
C19	0.0351 (17)	0.119 (4)	0.0399 (18)	−0.002 (2)	0.0073 (14)	−0.013 (2)
C20	0.0389 (18)	0.124 (4)	0.0366 (17)	0.018 (2)	0.0057 (14)	0.015 (2)
C21	0.0395 (16)	0.088 (3)	0.0434 (17)	0.0170 (17)	0.0171 (14)	0.0127 (17)
C22	0.0343 (14)	0.065 (2)	0.0340 (14)	0.0068 (14)	0.0142 (12)	0.0029 (13)

### Geometric parameters (Å, º)

**Table:**

Cl1—C18	1.736 (4)	C9—C10	1.486 (3)
Cl2—C21	1.738 (4)	C10—C15	1.389 (4)
S1—O1	1.425 (2)	C10—C11	1.391 (4)
S1—O2	1.4268 (19)	C11—C12	1.386 (4)
S1—N1	1.640 (2)	C11—H11	0.9500
S1—C1	1.759 (3)	C12—C13	1.375 (4)
O3—C7	1.333 (3)	C12—H12	0.9500
O3—H3O	0.8400	C13—C14	1.384 (4)
O4—C9	1.247 (3)	C13—H13	0.9500
N1—C8	1.440 (3)	C14—C15	1.384 (4)
N1—C16	1.491 (3)	C14—H14	0.9500
C1—C2	1.384 (4)	C15—H15	0.9500
C1—C6	1.397 (3)	C16—C17	1.506 (4)
C2—C3	1.386 (4)	C16—H16A	0.9900
C2—H2	0.9500	C16—H16B	0.9900
C3—C4	1.372 (4)	C17—C22	1.389 (4)
C3—H3	0.9500	C17—C18	1.404 (4)
C4—C5	1.379 (4)	C18—C19	1.387 (5)
C4—H4	0.9500	C19—C20	1.365 (6)
C5—C6	1.396 (4)	C19—H19	0.9500
C5—H5	0.9500	C20—C21	1.383 (5)
C6—C7	1.467 (4)	C20—H20	0.9500
C7—C8	1.368 (3)	C21—C22	1.386 (4)
C8—C9	1.448 (4)	C22—H22	0.9500
			
O1—S1—O2	119.62 (12)	C12—C11—C10	119.7 (3)
O1—S1—N1	107.33 (12)	C12—C11—H11	120.1
O2—S1—N1	107.70 (12)	C10—C11—H11	120.1
O1—S1—C1	108.03 (12)	C13—C12—C11	120.1 (3)
O2—S1—C1	110.19 (12)	C13—C12—H12	119.9
N1—S1—C1	102.62 (11)	C11—C12—H12	119.9
C7—O3—H3O	109.5	C12—C13—C14	120.5 (3)
C8—N1—C16	116.06 (19)	C12—C13—H13	119.7
C8—N1—S1	114.13 (16)	C14—C13—H13	119.7
C16—N1—S1	119.42 (18)	C13—C14—C15	119.8 (3)
C2—C1—C6	121.6 (2)	C13—C14—H14	120.1
C2—C1—S1	120.8 (2)	C15—C14—H14	120.1
C6—C1—S1	117.5 (2)	C14—C15—C10	120.0 (3)
C1—C2—C3	119.0 (3)	C14—C15—H15	120.0
C1—C2—H2	120.5	C10—C15—H15	120.0
C3—C2—H2	120.5	N1—C16—C17	113.8 (2)
C4—C3—C2	120.1 (3)	N1—C16—H16A	108.8
C4—C3—H3	120.0	C17—C16—H16A	108.8
C2—C3—H3	120.0	N1—C16—H16B	108.8
C3—C4—C5	121.2 (3)	C17—C16—H16B	108.8
C3—C4—H4	119.4	H16A—C16—H16B	107.7
C5—C4—H4	119.4	C22—C17—C18	118.0 (3)
C4—C5—C6	120.1 (3)	C22—C17—C16	119.1 (3)
C4—C5—H5	120.0	C18—C17—C16	122.9 (3)
C6—C5—H5	120.0	C19—C18—C17	120.7 (3)
C5—C6—C1	118.1 (2)	C19—C18—Cl1	118.4 (3)
C5—C6—C7	121.3 (2)	C17—C18—Cl1	120.8 (3)
C1—C6—C7	120.6 (2)	C20—C19—C18	120.3 (3)
O3—C7—C8	121.4 (2)	C20—C19—H19	119.9
O3—C7—C6	114.9 (2)	C18—C19—H19	119.9
C8—C7—C6	123.7 (2)	C19—C20—C21	119.9 (3)
C7—C8—N1	119.5 (2)	C19—C20—H20	120.0
C7—C8—C9	121.2 (2)	C21—C20—H20	120.0
N1—C8—C9	119.2 (2)	C20—C21—C22	120.4 (4)
O4—C9—C8	119.6 (2)	C20—C21—Cl2	120.1 (3)
O4—C9—C10	119.6 (2)	C22—C21—Cl2	119.4 (3)
C8—C9—C10	120.8 (2)	C21—C22—C17	120.5 (3)
C15—C10—C11	119.9 (3)	C21—C22—H22	119.7
C15—C10—C9	119.4 (2)	C17—C22—H22	119.7
C11—C10—C9	120.5 (2)		
			
O1—S1—N1—C8	−61.89 (19)	S1—N1—C8—C9	138.3 (2)
O2—S1—N1—C8	168.09 (17)	C7—C8—C9—O4	−16.1 (4)
C1—S1—N1—C8	51.8 (2)	N1—C8—C9—O4	161.9 (2)
O1—S1—N1—C16	154.46 (18)	C7—C8—C9—C10	162.6 (2)
O2—S1—N1—C16	24.5 (2)	N1—C8—C9—C10	−19.5 (3)
C1—S1—N1—C16	−91.83 (19)	O4—C9—C10—C15	−36.4 (4)
O1—S1—C1—C2	−96.7 (2)	C8—C9—C10—C15	144.9 (3)
O2—S1—C1—C2	35.6 (3)	O4—C9—C10—C11	138.0 (3)
N1—S1—C1—C2	150.1 (2)	C8—C9—C10—C11	−40.7 (4)
O1—S1—C1—C6	80.8 (2)	C15—C10—C11—C12	0.3 (4)
O2—S1—C1—C6	−146.9 (2)	C9—C10—C11—C12	−174.1 (3)
N1—S1—C1—C6	−32.4 (2)	C10—C11—C12—C13	−0.7 (4)
C6—C1—C2—C3	−0.8 (4)	C11—C12—C13—C14	0.0 (4)
S1—C1—C2—C3	176.6 (2)	C12—C13—C14—C15	1.1 (5)
C1—C2—C3—C4	0.3 (4)	C13—C14—C15—C10	−1.5 (4)
C2—C3—C4—C5	0.3 (5)	C11—C10—C15—C14	0.8 (4)
C3—C4—C5—C6	−0.5 (5)	C9—C10—C15—C14	175.3 (3)
C4—C5—C6—C1	0.0 (4)	C8—N1—C16—C17	−62.6 (3)
C4—C5—C6—C7	−179.3 (3)	S1—N1—C16—C17	80.4 (3)
C2—C1—C6—C5	0.7 (4)	N1—C16—C17—C22	−86.3 (3)
S1—C1—C6—C5	−176.8 (2)	N1—C16—C17—C18	92.3 (3)
C2—C1—C6—C7	−180.0 (2)	C22—C17—C18—C19	2.1 (4)
S1—C1—C6—C7	2.5 (3)	C16—C17—C18—C19	−176.5 (3)
C5—C6—C7—O3	16.2 (4)	C22—C17—C18—Cl1	−177.5 (2)
C1—C6—C7—O3	−163.1 (2)	C16—C17—C18—Cl1	3.9 (4)
C5—C6—C7—C8	−166.5 (2)	C17—C18—C19—C20	−0.7 (5)
C1—C6—C7—C8	14.2 (4)	Cl1—C18—C19—C20	178.9 (3)
O3—C7—C8—N1	−175.6 (2)	C18—C19—C20—C21	−1.2 (5)
C6—C7—C8—N1	7.3 (4)	C19—C20—C21—C22	1.8 (5)
O3—C7—C8—C9	2.3 (4)	C19—C20—C21—Cl2	−177.8 (3)
C6—C7—C8—C9	−174.8 (2)	C20—C21—C22—C17	−0.4 (4)
C16—N1—C8—C7	101.2 (3)	Cl2—C21—C22—C17	179.2 (2)
S1—N1—C8—C7	−43.8 (3)	C18—C17—C22—C21	−1.5 (4)
C16—N1—C8—C9	−76.8 (3)	C16—C17—C22—C21	177.1 (2)

### Hydrogen-bond geometry (Å, º)

**Table:**

*D*—H···*A*	*D*—H	H···*A*	*D*···*A*	*D*—H···*A*
C3—H3···O4^i^	0.95	2.60	3.310 (4)	132
O3—H3*O*···O4	0.84	1.80	2.539 (3)	146
C11—H11···N1	0.95	2.62	3.003 (3)	105
C16—H16*A*···O2	0.99	2.45	2.862 (3)	105
C16—H16*B*···Cl1	0.99	2.67	3.120 (3)	108

Symmetry code: (i) *x*, *y*+1, *z*.
